# Land use/land cover change, physico-chemical parameters and freshwater snails in Yewa North, Southwestern Nigeria

**DOI:** 10.1371/journal.pone.0246566

**Published:** 2021-02-08

**Authors:** Opeyemi G. Oso, Alex B. Odaibo

**Affiliations:** Department of Zoology, Parasitology Research Unit, University of Ibadan, Ibadan, Nigeria; Johns Hopkins University, UNITED STATES

## Abstract

The management of ecosystem has been a major contributor to the control of diseases that are transmitted by snail intermediate hosts. The ability of freshwater snails to self-fertilize, giving rise to thousands of hatchlings, enables them to contribute immensely to the difficulty in reducing the endemicity of some infections in the world. One of the effects of land use/land cover change (LU/LCC) is deforestation, which, in turn, leads to the creation of suitable habitats for the survival of freshwater snails. This study was aimed at studying the land use/land cover change, physico-chemical parameters of water bodies and to understand the interplay between them and freshwater snails in an environment where a new industrial plant was established. Landsat TM, 1984, Landsat ETM+ 2000 and Operational land Imager (OLI) 2014 imageries of the study area were digitally processed using ERDAS Imagine. The land use classification includes settlement, water bodies, wetlands, vegetation and exposed surface. Dissolved oxygen, water temperature, pH, total dissolved solids and conductivity were measured with multipurpose digital meters. Snail sampling was done at each site for 30 minutes along the littoral zones, using a long-handled scoop (0.2mm mesh size) net once every month for 24 months. Independent *t*-test was used to determine the variation between seasons, Spearman’s rank correlation coefficient was used to test the relationship between physico-chemical parameters and snail species while regression was used to analyze the relationship between LU/LCC and freshwater snails. Species’ richness, diversity and evenness were examined using Margalef, Shannon Weiner and Equitability indexes. Snail species recovered include: *Bulinus globosus*, *Bulinus jousseaumei*, *Bulinus camerunensis*, *Bulinus senegalensis*, *Bulinus forskalii*, *Amerianna carinatus*, *Ferrissia* spp., *Segmentorbis augustus*, *Lymnaea natalensis*, *Melanoides tuberculata*, *Physa acuta*, *Gyraulus costulatus*, *Indoplanorbis exuxtus* and *Gibbiella* species. Out of the total snails recovered, *M*. *tuberculata* (2907) was the most abundant, followed by *Lymnaea natalensis* (1542). The highest number of snail species was recovered from Iho River while the least number of snails was recovered from Euro River. The mean and standard deviation of physico-chemical parameters of the water bodies were DO (2.13±0.9 mg/L), pH (6.80±0.4), TDS (50.58±18.8 mg/L), Temperature (26.2±0.9°C) and Conductivity (74.00±27.5 μS/cm). There was significant positive correlation between pH and *B*. *globosus* (r = 0.439; P<0.05). Dissolved oxygen showed significant positive correlation with *B*. *globosus* (r = 0.454; P<0.05) and *M*. *tuberculata* (r = 0.687; P<0.01). There was a positive significant relationship between LULCC and *B*. *camerunensis* (p<0.05). The positive relationship between LULCC and the abundance of *B*. *globosus*, *B*. *jousseaumei* was not significant. The area covered by water bodies increased from 3.72 to 4.51 kilometers; this indicates that, more suitable habitats were being created for the multiplication of freshwater snails. We therefore conclude that, increase in areas suitable for the survival of freshwater snails could lead to an increase in water-borne diseases caused by the availability of snail intermediate hosts.

## Introduction

Land use/land cover change (LU/LCC) is the most significant regional anthropogenic disturbance to the environment [1]. Land use and land cover change often occurs as a result of strong interaction between natural and anthropogenic activities. Land use/land cover change is driven by some underlying factors which are central to environmental processes and management, through their influence on biodiversity, heat, moisture contents, trace gas emissions, carbon cycling, livelihoods, ecological processes and a wide range of socio-economic practices [2,3]. Therefore, understanding the concept of land use/land cover dynamics is of utmost importance in order to examine various ecological and developmental consequences of land use change over a space of time in an area. The result of land use/land cover helps policy makers to channel limited resources in the right direction for optimum development in their regions [3].

In science, land cover and land use are often studied in association with each other. Remote sensing, satellite imagery and aerial photography can identify land cover but inferring land use often requires more knowledge of the study region through ground truthing. Land use/land cover change occurs at global, national and local scales. The growth in world human population has a huge potential for changing the face of the earth surface and knowledge on land use and land cover is the basis on which the past and present human interactions and the impacts of such interactions with natural resources and the environment can be understood. Biodiversity and ecosystem functioning is gradually reducing because of the effect of land use/land cover changes over the years. Anthropogenic disturbances can alter ecosystems up to several kilometers [4,5], thereby affecting a significant portion of land surface structure [6]. These disturbances can lead to high prevalence of diseases due to movement of snail intermediate hosts, vectors and hosts as well as providing suitable environmental conditions [7,8]. Multiple factors are involved in land use/land cover change processes and these vary across regions and time [9]. The creation of industries and agricultural expansion through deforestation is one of the proximate causes of land use/land cover change [10]. Excessive vegetation cover leads to the spread of infectious diseases in temperate regions [11,12]. Approximately one-third of the world landscapes is being used for industrial activities or growing crops [13]. Major changes in human activities, particularly through large-scale agriculture and creation of industries have been identified as the major cause of the dramatic change in land cover and land use patterns globally. Usually land cover and land use changes takes a very long time before the effect can be felt in the past but now, the effect is being felt within a very short interval.

The increasing concern for the management of natural resources in recent times have been necessitated by the increasing demographic pressures and its associated man-made activities which have led to serious environmental stress and ecological instability. The effects of land use/land cover changes over 300 years have taken significant dimension [14]. Land use/land cover change has been found to be more dominant in developing countries, due to the high propensity of population growth rate and the subsequent resource over-exploitation. The impacts of these environmental problems are serious; they include food security, human vulnerability, health effects and threatened earth surface [15].

Schistosomiasis is one of the diseases that are triggered by LU/LCC. The removal of vegetation cover changes the ecology of freshwater snail species populations by increasing sunlight penetration, encouraging growth of vegetation, and changing water levels and flow rates with respect to different ecological zones. However, snail species do not survive these changes, but those that survive tend to be better hosts for the parasitic worms [16]. Freshwater snails of medical importance continue to play a significant role in the transmission of schistosomiasis worldwide [17]. In Nigeria, *Bulinus* and *Biomphalaria* species are intermediate hosts of *Schistosoma haematobium* and *Schistosoma mansoni* respectively and they can be found in a wide range of freshwater habitats [18,19]. Snail species inhabit water bodies with a wide range of dissolved chemical contents and their abundance is dependent on water chemical content. Generally, rainfall among other factors affect the distribution of freshwater snails in different areas [19]. Water temperature, pH, dissolved oxygen, and conductivity have effects on the fecundity, mortality and death of planorbids [20,21]. Other factors that affect the distribution of freshwater snails include light, water velocity, vegetation, water depth [22,23]. Schistosome infections in humans and snail intermediate hosts have been reported [24]. However, large scale relationship between freshwater snails and physico-chemical parameters in relation to land use/land cover change due to anthropogenic activities is essential. This study investigated the interplay between population dynamics of freshwater snails and physico-chemical parameters, over a two-year study period and the potential effects of land use/land cover change in the spread of freshwater snails.

## Materials and methods

This study was conducted in Yewa North Local Government Area (YNLGA), Ogun State between January 2013 and December 2014. The Yewa River divides into different tributaries across different villages in the Local Government Area. It was observed that one of largest industrial plant was newly constructed in the study area. Few studies in these areas reported high prevalence of schistosomiais [24,25]. Most inhabitants of these communities depend on river for most of their activities including drinking, washing, farming and other recreational activities. The limited pipe borne water located in these areas has been reported to be hard; therefore people living in the communities prefer to use water from these river for most of their activities.

Snail sampling was done at monthly intervals for twenty-four months in twenty sampling stations. Snails were collected according to the method described by Olofinoye and Odaibo [18]. Snail sampling was done with the aid of scoop (0.2mm mesh size) net. The scoop was carried out on site and searched for snails for 30 minutes; recovered snails were kept in perforated lid pre-labelled containers. The snails were identified according to the guidelines provided by Brown and Kristensen [26]. Infections in snail species were performed by our research group and the results were presented elsewhere [27]. Water conductivity, Total Dissolved Solids, and hydrogen ion concentration were quantified at each site using portable electronic multipurpose meter (Extech Instrument Corp. Waltham, MA) and pH meter (CE Portable pH meter). Dissolved oxygen was measured using YSI 550A DO meter.

### GIS/RS data processing

Geo-referencing of the topographical map, digitizing the map for easy identification of the features, image Enhancement, Image re-sampling, Image classification, and Image interpretation were performed using QGIS. The study applied remotely sensed imagery, digital image processing and geographic information systems techniques to analyze land use and land cover change around Yewa North Local Government Area. This involves the generation of land use and land cover statistics for 1984, 2000 and 2014 in a manner suitable for change detection analysis using Landsat imageries. Supervised method was used for the classifications. Supervised classification was used to cluster pixels in a data set based on ground truth survey, with the user-defined training classes [28]. The images were processed, interpreted and classified using ERDAS, while the final map of the study area was completed in QGIS. The land uses were classified into vegetation (V), settlement (STL), water body (WB), exposed surface and wetland (WL).

### Land use and land cover classification scheme

The Landsat data were acquired from the global land-cover host (Source: U.S. Geological Survey). The images were Enhance Thematic Mapper plus (ETM^+^) image 1984, 2000 and Operational land Imager (OLI) 2014. A false Colour Composite operation was performed using the Idrisi software and the landsat bands were combined in the order of band 4, band 3 and band 2 for landsat TM and ETM+ while landsat OLI was composited in the order of band 4 and band 3 due to change in sensor. The False Color Composite was further classified using the maximum likelihood classification technique. A supervised classification was performed by creating a training sample and based on spectral signature curve, various land use classes were created namely; Settlements; Exposed surfaces; wetlands, water body and vegetation. The classified map was generated for years 1984; 2000 and 2014 respectively. Post classification comparison of classified LU/LC statistics was carried out using the cross tabulation method for assessing the changes of the various classes in the LU/LC. The statistics of the LU/LC was generated separately alongside the classified images for each year.

### Data analysis

All data were entered into an excel Spreadsheet, carefully checked for errors and transferred into Statistical Package for Social Science (SPSS Inc. Chicago, USA) for analysis. Independent *t*-test was used to determine variations between seasons, Spearman’s rank correlation coefficient was used to test the relationship between freshwater snail abundance and physico-chemical parameters of the river bodies while regression was used to analyze the relationship between LU/LCC and freshwater snails. Species’ richness, diversity and evenness were examined using Margalef, Shannon Weiner and Equitability indexes. The P-values <0.05 were considered statistically significant.

### Ethics approval

We obtained an approval to carry out this study from Ogun State ministry of health. We also got an approval from the village heads.

## Results

A total of 9,373 gastropods representing eighteen species, six orders and eight families were recovered from the water bodies during the study period (Table 1). Out of the total snails recovered, *M*. *tuberculata* (2907) was the most abundant, followed by *Lymnaea natalensis* (1542). The abundance of *B*. *globosus*, *B*. *jousseaumei*, *B*. *camerunensis*, *B*. *senegalensis* and *B*. *forskalii* were 1005, 481, 154, 71 and 29 respectively. The highest number of snail species was recovered from Iho River while the least number was recovered from Euro River. No snail was collected from Ajerogun River in Ibese community throughout the sampling period. *Amerianna carinatus* was found in all the sampling sites except Joga orile, Iboro and Ibese while *L*. *lybicus* was also found in most sampling sites but not in Ibese and Ijale ketu communities. In all, snail abundance in each site was significantly different across the sampling locations (P<0.5). Generally, snail abundance was slightly higher in rainy season with a total of 4716 snails while a total of 4657 snails were recovered in dry season; the variation was statistically significant (P<0.05).

**Table 1:** Abundance of freshwater snail species.

Snail species																				
Location	*A*.*c*	*B*.*c*	*B*.*g*	*B*.*j*	*B*.*s*	*B*.*p*	*F*. spp	*G*.*c*	*I*.*e*	*L*.*l*	*L*.*n*	*M*.*t*	*P*.*a*	*S*.*a*	*G*. spp	*B*.*f*	*A*.*w*	*P*.*m*	Total	% Abundance
**Bareke Ayetoro**	23	46	95	136	42	0	9	18	0	91	6	169	0	4	0	23	0	3	665	7.09
**Orori Ayetoro**	44	38	24	11	4	0	8	20	0	108	65	0	16	7	0	0	8	0	353	3.77
**Iju Joga orile**	0	0	0	0	0	0	4	7	0	148	0	165	0	1	0	0	0	1	326	3.48
**Ikiso Sawonjo**	168	19	0	0	1	0	39	10	0	21	6	36	0	0	0	0	0	0	300	3.20
**Iju Iboro**	0	0	0	0	0	0	6	1	0	294	0	0	0	0	0	0	0	0	301	3.21
**Iju Imasayi**	7	1	270	141	2	0	1	50	1	47	392	5	6	2	0	0	68	0	993	10.59
**Iniya Maria**	29	0	0	0	0	0	1	1	0	137	0	0	0	0	0	0	0	0	168	1.79
**Balogun**	1	0	25	7	0	0	0	9	0	29	7	1	0	3	62	0	0	0	144	1.54
**Ajerogun Ibese**	0	0	0	0	0	0	0	0	0	0	0	0	0	0	0	0	0	0	0	0.00
**Iju Igbogila**	18	5	18	10	0	0	17	16	0	12	65	0	1	1	0	0	4	0	167	1.78
**Euro Eggua**	1	0	0	0	0	0	1	13	0	53	10	0	0	2	0	0	0	0	80	0.85
**Yewa Eggua**	9	0	2	0	0	0	42	80	0	31	9	0	0	0	20	0	0	0	193	2.06
**Idi Eggua**	44	9	138	41	2	0	10	42	1	128	18	1	0	0	0	0	0	0	434	4.63
**Idi Agbon**	106	10	96	11	5	0	11	24	0	6	0	0	0	3	0	0	0	0	272	2.90
**Iho Ibayun**	1	0	0	0	0	0	73	22	0	105	26	2528	0	0	0	0	0	5	2760	29.45
**Yewa Igan Alade**	4	4	129	19	9	0	19	23	0	12	32	0	0	2	0	0	0	0	253	2.70
**Yewa Owode**	54	10	85	66	0	0	36	72	59	14	21	1	5	7	0	6	0	0	436	4.65
**Iju Ijale ketu**	29	12	79	12	4	1	0	7	2	0	631	0	10	2	0	0	0	0	789	8.42
**Isopa Ijoun**	26	0	3	2	2	0	21	58	0	24	223	1	0	1	0	0	0	0	361	3.85
**Idi Ijoun**	1	0	40	25	0	0	10	103	0	33	14	0	0	0	0	0	0	0	226	2.41
**Total**	**566**	**154**	**1005**	**481**	**71**	**1**	**329**	**579**	**63**	**1399**	**1542**	**2907**	**38**	**35**	**85**	**29**	**80**	**9**	**9373**	**100**

A.*c-Ameriana carinatus*, *B*.*c-Bulinus camerunensis*, *B*.*g-Bulinus globosus*, *B*.*s- Bulinus senegalensis*, *B*.*p- Biomphalaria pfeifferi*, *F*.sp.*- Ferrissia* Species, *G*.*c- Gyraulus costulatus*, *I*.*e- Indoplanorbis exustus*, *L*.*l- Lanistes lybicus*, *L*.*n-Lymnaea natalensis*, *M*.*t- Melanoides tuberculata*, *P*.*a- Physa acuta*, *S*.*a- Segmentorbis augustus*, *G*.sp.*- Gabbiella* Species, *B*.*f- Bulinus forskalii*, *A*.*w-Aplexa waterloti*, *P*.*m-Potadoma moerchi*.

*Bulinus globosus* forms significant positive correlation with *B*. *jousseaumei* (r = 0.642; P<0.01), *Ferrissia* sp. (r = 0.420; P<0.05), *G*. *costulatus* (r = 0.523, P<0.01), *I*. *exustus* (r = 0.577; P<0.01), *L*. *lybicus* (r = 0.585; P<0.01), *L*. *natalensis* (r = 0.462, P<0.05), *Melanoides tuberculata* (r = 0.555; P<0.01). However, *B*. *globosus* correlated negatively with *P*. *acuta* (r = -392; P>0.05), *S*. *augustus* (r = -0.098; P>0.05) and *A*. *waterloti* (r = -0.295; P>0.05). *Bulinus jousseaumei* significantly correlated with *Gyraulus costulatus* (r = 0.959; P<0.595), *I*. *exustus* (r = 0.674; P<0.01), *Lanistes lybicus* (r = 0.654; P<0.01) and *L*. *natalensis* (r = 0.615; P<0.01). *Ferrissia* sp. correlated positively with *G*. *costulatus* (r = 0.673; P<0.01), *I*. *exustus* (r = 0.511; P<0.05), *L*. *lybicus* (r = 0.583; P<0.01) and *M*. *tuberculata* (r = 0.504; P<0.05). *G*. *costulatus* also forms significant positive correlation with *I*. *exustus* (r = 0.523; P<0.01), *L*. *lybicus* (r = 0.779; P<0.01) and *M*. *tuberculata* (r = 0.431; P<0.05). *I*. *exustus* correlated positively with *L*. *lybicus* (r = 0.543; P<0.01), *L*. *natalensis* (r = 0.457; P<0.05) and *M*. *tuberculata* (r = 0.458; P<0.05). *L*. *natalensis* correlated positively with *L*. *lybicus* (r = 0.545; P<0.01), however, *L*. *natalensis* correlated negatively with *A*. *waterloti* (r = -0.430; P<0.05). *M*. *tuberculata* showed significant negative relationship with *P*. *acuta* (r = -0.481; P<0.05) and *A*. *waterloti* (r = -0.437; P<0.05), however, significant positive relationship occurred between *M*. *tuberculata* and *Gabbiella* sp. (r = 0.532; P<0.01).

The lowest DO value was recorded in Bareke River in Ayetoro community with mean and range value of 0.82±0.31 (0.13–1.57) mg/L while the highest value was recorded in Eggua community with mean and range value of 2.83±1.69 (0.42–5.75) mg/L. The dissolved oxygen varied across different locations during the sampling period (Table 2). Generally, there was no significant variation in DO between seasons. Dissolved oxygen showed significant positive correlation with *B*. *globosus* (r = 0.454; P<0.05), *M*. *tuberculata* (r = 0.687; P<0.01) and *Gabbiella* sp. (r = 0.488; P<0.05). However, DO showed significant negative correlation with *A*. *waterloti* (r = -0.623; P<0.01) and *S*. *augustus* (r = -0.502; P<0.05).

**Table 2:** Mean and range values of physico-chemical characteristics of water bodies.

Water contact sites	DO (mg/L)	pH	CONDUCTIVITY (μS/cm)	TDS (mg/L)	TEMPERATURE (°C)
**Bareke Ayetoro**	0.82±0.31(0.13–1.57)	6.69±0.31(6.11–7.40)	84.3±34.70(30.5–187.70)	52.46±17.30(20.4–94.3)	25.9±0.0.83(24.20–27.4)
**Orori Ayetoro**	0.83±0.23(0.50–1.55)	6.79±0.38(6.20–7.80)	92.69±27.38(44.5–141.30)	63.61±22.60(29.8–120.6)	25.74±0.83(24.40–27.4)
**Iju Joga orile**	1.00±0.42(0.53–2.16)	6.41±0.37(5.61–7.39)	52.28±12.10(26.40–77.60)	36.00±10.19(17.70–54.60)	26.21±1.21(23.90–28.90)
**Ikiso Sawonjo**	1.95±0.58(0.93–3.06)	6.69±0.40(6.04–7.78)	51.16±11.77(28.60–80.40)	35.16±11.24(14.00–56.70)	25.72±0.84(24.10–27.20)
**Iju Iboro**	3.85±1.42(0.56–5.28)	6.82±0.52(6.06–8.44)	48.22±22.58(11.30–95.10)	33.32±15.37(6.00–66.50)	25.80±0.87(24.00–27.50)
**Iju Imasayi**	2.43±0.91(0.54–3.75)	6.80±0.35(6.08–7.50)	64.51±25.14(33.20–149.30)	43.89±16.93(22.20–100)	26.15±1.12(24.00–28.40)
**Iniya Maria**	2.88±0.97(0.66–4.16)	6.85±0.52(6.10–8.43)	65.74±18.79(34.70–98.80)	46.99±17.99(23.20–89.70)	26.12±0.86(24.00–27.80)
**Balogun**	1.15±0.66(0.30–2.88)	6.71±0.35(6.10–7.45)	132.64±48.96(45.40–226.00)	91.11±35.97(30.40–158.00)	26.40±1.50(24.00–29.60)
**Ajerogun Ibese**	1.05±0.37(0.44–2.17)	5.47±0.46(5.00–7.30)	58.65±13.51(34.80–89.60)	41.900±11.45(23.30–75.90)	27.26±0.69(25.80–28.60)
**Iju Igbogila**	2.12±1.05(0.59–3.87)	6.86±0.42(6.20–7.78)	68.50±16.04(43.00–101.10)	44.14±10.35(24.50–69.70)	26.57±0.86(24.20–28.20)
**Euro Eggua**	2.52±1.27(0.47–4.02)	6.98±0.44(6.20–7.73)	72.73±20.65(40.70–123.20)	50.23±15.92(27.30–90.60)	26.88±1.62(24.30–32.30)
**Yewa Eggua**	2.83±1.69(0.42–5.75)	7.15±0.49(6.10–7.93)	77.54±15.74(52.40–112.60)	54.30±12.12(35.10-80-80)	26.75±0.93(25.00–28.40)
**Idi Eggua**	2.64±1.36(0.48–4.93)	7.02±0.53(5.60–7.80)	58.19±15.73(30.60–94.00)	40.01±10.39(20.50–63.00)	26.10±1.14(24.50–29.50)
**Idi Agbon**	1.68±1.20(0.33–5.34)	6.67±1.50(6.00–7.90)	153.89±48.28(106.30–244.00)	105.09±34.09(71.40–171.00)	25.10±5.51(24.30–29.60)
**Iho Ibayun**	3.06±1.56(0.44–5.23)	7.00±0.40(6.40–7.95)	92.05±25.17(55.60–156.20)	65.01±16.87(37.30–102.40)	23.48±0.80(23.90–26.90)
**Yewa Igan Alade**	2.66±1.67(0.48–5.47)	7.11±0.56(6.38–8.30)	85.86±24.56(40.00–148.20)	58.93±20.47(26.80–124.30)	26.55±1.14(24.70–29.00)
**Yewa Owode**	2.69±1.77(0.47–5.64)	7.17±0.54(6.20–8.04)	81.99±19.33(42.90–127.00)	56.80±14.46(28.70–89.10)	26.20±1.08(24.10–29.50)
**Iju Ijale ketu**	0.87±0.40(0.28–1.67)	6.66±0.37(6.02–7.49)	63.92±28.05(24.50–140.50)	43.01±18.67(16.40–96.60)	25.55±4.08(6.72–27.800
**Isopa Ijoun**	2.18±1.17(0.42–4.45)	7.02±0.50(6.20–8.02)	44.85±12.37(23.70–75.40)	31.21±6.71(16.10–48.00)	26.97±1.08(25.50–29.60)
**Idi Ijoun**	2.50±1.50(0.41–4.99)	7.00±0.39(6.45–7.64)	51.52±15.11(24.10–82.70)	35.27±10.13(18.20–55.40)	26.29±1.88(18.60–28.20)

The variation in pH across all the sampling points was minimal. The highest pH value was recorded in Iju River with mean and range value of 6.82±0.52 (6.06–8.44) while the least value was recorded in Ajerogun River with mean and range value of 5.47±0.46 (5.00–7.7.30); besides, most of the pH measures in Ajerogun River were below 6.0. Though pH correlated negatively with *A*. *waterloti* (r = -0.499; P<0.05); however, it correlated positively with *B*. *globosus* (r = 0.439; P<0.05), *B*. *jousseaumei* (r = 0.721; P<0.01), *L lybicus* (r = 0.438, P<0.05) and *L*. *natalensis*. (r = 0.620; P<0.01).

Conductivity values were moderate in most of the water contact sites except in some few sampling sites where the conductivity values were high. The highest conductivity value was recorded in Idi River in Agbon community with mean and range value of 153.89±48.28(106.30–244.00) μS/cm while the least conductivity value was recorded in Iju River in Iboro community with mean and range value of 48.22±22.58 (11.30–95.10) μS/cm. There was a negative relationship between conductivity and most of the freshwater snails, however, the negative correlation between conductivity and *Aplexa waterloti* was significant (r = -0.456; P<0.05).

Total dissolved solids (TDS) varied across all the sampling sites. The lowest value of TDS was recorded in Iju River in Iboro community with mean and range value of 33.32±15.37 (6.00–66.50) mg/L while the highest TDS value was recorded in Idi River in Agbon community with mean and range value of 105.09±34.09 (71.40–171.00) mg/L. TDS forms significant negative correlation with *Gyraulus costulatus* (r = -0.412; P<0.05) mg/L and *A*. *waterloti* (r = -0.453; P<0.05) mg/L.

Temperature varied in all the sampling locations during the sampling period. The highest temperature was recorded in Euro River in Eggua community with mean and range values of 26.88±1.62 (24.30–32.30) °C while the least was recorded in Idi River in Ijoun community with mean and range value of 26.29±1.88 (18.60–28.20) °C. There was a significant negative correlation between temperature and *Ferrissia* sp. (r = -0.528; P<0.01), *L*. *lybicus* (r = -0.496; P<0.05), *G*. *costulatus* (r = -0.432; P<0.05); however, temperature forms significant positive correlation with *P*. *acuta* (r = 0.452; P<0.452). The relationship between temperature and the following snail species: *A*. *carinatus* (r = -0.279; P>0.05), *B*. *camerunensis* (r = -0.048; P>0.05), *B*. *globosus* (r = -0.343; P>0.05), *B*. *jousseaumei* (r = -0.125; P>0.05), *B*. *senegalensis* (r = -0.062; P>0.05), *I*. *exustus* (r = -0.064; P>0.05), *L*. *natalensis* (r = -0.216; P>0.05), *M*. *tuberculata* (r = -0.143; P>0.05), *S*. *augustus* (r = -0.286; P>0.05), *Gabbiella* sp. (r = -0.084; P>0.05), *A*. *waterloti* (r = -0.063; P>0.05), *P*. *moerchi* (r = -0.154; P>0.05) was not significant. The recovered snail species diversity (0.943), richness (1.974) and evenness (0.846) were highest in Yewa River (Table 3).

**Table 3:** Biodiversity indices of snail species.

Water contact sites	Shannon index (H’)	Shannon-Wiener Diversity index (H)	Evenness Index (E)	Margalef’s Species Richness (d)
**Bareke Ayetoro**	2.093	0.909	0.816	1.846
**Orori Ayetoro**	2.067	0.898	0.832	1.875
**Iju Joga orile**	0.875	0.380	0.488	0.864
**Ikiso Sawonjo**	1.416	0.615	0.681	1.227
**Iju Iboro**	0.120	0.052	0.109	0.350
**Iju Imasayi**	1.615	0.701	0.612	1.884
**Iniya Maria**	0.531	0.230	0.383	0.585
**Balogun**	1.606	0.698	0.731	1.610
**Ajerogun Ibese**	0.000	0.000	0.000	0.000
**Iju Igbogila**	1.918	0.833	0.800	1.954
**Euro Eggua**	1.030	0.447	0.575	1.141
**Yewa Eggua**	1.559	0.677	0.801	1.140
**Idi Eggua**	1.757	0.763	0.733	1.647
**Idi Agbon**	1.537	0.668	0.700	1.427
**Iho Ibayun**	0.398	0.173	0.204	0.757
**Igan Alade**	1.644	0.714	0.714	1.626
**Yewa Owode**	2.171	0.943	0.846	1.974
**Iju Ijale ketu**	0.821	0.356	0.342	1.499
**Isopa Ijoun**	1.256	0.546	0.546	1.528
**Idi Ijoun**	1.523	0.662	0.783	1.107

**Fig 1:**
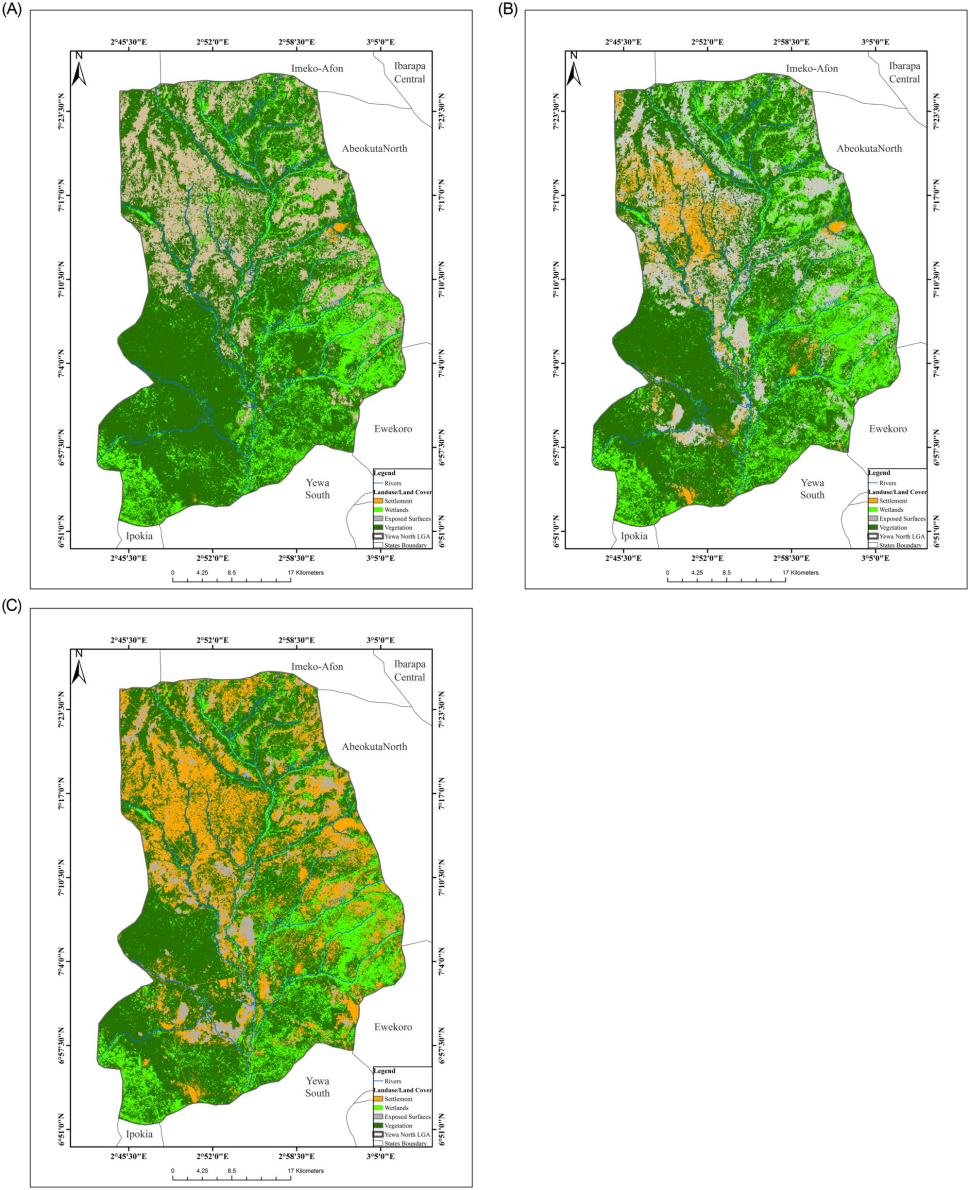
Land use/land cover (LULC) map of YNLGA. (A) Vegetation and Wetlands were more observed in year 1984. (B) Exposed Surfaces has increased due to high farming activities in year 2000. (C) Settlements has increased due to influx of people and developmental projects in year 2014.

Fig 1 shows the land use/land cover of Yewa North Local Government Area (YNLGA). Part of the North and East were wetlands while vegetation covered the Southern areas. The percentage of settlement increased from 3.33% to 42.62% in year 2014; however, there was a reduction in percentage of exposed surface in the study area (Table 4). The area covered by water bodies also increased from 3.72km to 4.51km (Table 5). Settlements were found scattered around YNLGA with the highest settlement found in Ayetoro. There was a significant positive relationship between LU/LC and abundance of *B*. *camerunensis* (p<0.05). Also, positive relationship which occurred between LU/LC and the abundance of *B globosus*, *B*. *jousseaumei* was not significant (p>0.05).

**Table 4:** Percentage of land use/land cover classes (1984–2014).

Land use/Land cover	Year 1984	Year 2000	Year 2014
Settlements	3.33	17.3	42.62
Wetlands	20.2	21.83	18.07
Vegetation	35.91	27.3	20.99
Exposed Surface	40.37	33.4	18.09
Water body	0.19	0.22	0.23

**Table 5:** Area in kilometer square of land use/land cover classes (1984–2014).

Land use/Land cover	Year 1984	Year 2000	Year 2014
Settlements	65.23	338.88	834.85
Wetlands	395.68	427.61	353.96
Vegetation	703.41	534.76	411.16
Exposed Surface	790.77	654.25	354.35
Water body	3.72	4.31	4.51

## Discussion

There was variation in the overall snail abundance and population density fluctuates between sites. Previous studies in Africa have associated such variation with differences between sites in vegetation types [22,29], substratum [30], and the presence or absence of other freshwater snail species [31,32]. Other important sources of variation between sites are local rainfall, seasonal water flows and water temperature [33]. From this study, it was apparent that the snail intermediate host populations undergo marked seasonal variation in abundance, although patterns differ between species, habitats and sites; this is in consonance with other similar studies [22,34]. Low snail densities during the peak rainy periods have been attributed to the flushing out of snails due to flooding of water bodies [34,35].

Environmental factors that influence snail distribution often vary considerably from site to site, even within short distances [36]. Photosynthesizing micro-organisms, high oxygen concentrations in water bodies and narrow boundary layers are factors that maintain an adequate supply of oxygen for development. However, anthropogenic eutrophication, enhanced by riverine runoff of fertilizers and the burning of fossil fuels [37], increases primary production in river bodies, thereby resulting in an accumulation of particulate organic matter and high microbial activity that consumes dissolved oxygen (DO) in bottom waters [37]. Low oxygen concentrations in water and weak velocity gradients retard or arrest development of micro-organisms. All these conditions which influence oxygen supply, could affect the evolution of the sizes and shapes of egg masses, embryos as well as adult snail species in the surrounding water [38,39]. The developmental delays can however be reversed or prevented entirely with supplemental oxygen [40,41].

The highest value of dissolved oxygen recorded in Yewa River in Eggua community might be due to fast flowing River types, washing away most of the pollutants [42]. Similar findings of high dissolved oxygen have also been reported in Ethiope River [43,44]. The low dissolved oxygen concentration recorded in Yewa River (Ijale Ketu) agreed with values reported for other Nigerian waters [45]. The low dissolved oxygen values revealed anoxic or septic condition during the dry season within the study period. Such low oxygen saturation has been reported in River Kaduna in dry season when there was little or no flow of water [46,47]. The low level of dissolved oxygen recorded is an indication of deteriorating water quality and it is probably as a result of death and decay of aquatic macrophytes, increased active organic decomposition in the bottom sediment and the absence of flow-induced turbulence, which ought to enhance oxygen dissolution in water [48]. The significant positive correlation of dissolved oxygen and some freshwater snails observed in this study have also been reported [49].

Lack of relationship between conductivity and all bulinids species is in agreement with similar study [50]. However, our report is in deviance with other studies which recorded negative relationship between snail intermediate hosts and conductivity [51]. Conductivity has been considered as an indicator or factor which may limit the distribution of snail species. Different species of snails live in biotopes with wide differences in conductivity. In a study conducted in Liberia, *B*. *globosus* was found in water with low conductivity [52] while in other African countries, bulinids were found in biotopes with high conductivity [53,54]. Tolerance to conductivity varies with different species of snails and stages of development. Egg masses and hatchlings are more sensitive to high conductivity than adults [55]. In this study, the recorded values of conductivity varied between 11.30 and 244 μS/cm across different sampling locations. Conductivity was observed to increase during the dry season and decreased in the rainy season. The increase in conductivity during the dry season was probably due to the high evaporation.

Extreme weather conditions such as summer heat waves were predicted to become more frequent in the future due to global climate change [56]. This was suggested to have considerable impacts on natural populations and communities of organisms, thereby changing species interaction with the environment [57,58]. Roughly, the optimal temperature range for *Bulinus* sp. and *Biomphalaria* sp. development is between 20°C and 30°C, with thermal death occurring at temperatures either below 16°C or above 40°C [59]. Unpredictable occurrence of extreme temperatures can have deleterious effects on organisms [60,61]. Furthermore, when temperature becomes too extreme, enzymatic function, membrane structure, metabolic rate and oxygen supply can be impaired [62,63]. In addition, exposure to high temperature may lead to a higher requirement for micronutrients (e.g., zinc, copper, vitamins) [64,65] as they are involved in response and tolerance mechanisms [66]. The negative correlation between temperature and all bulinids in this study was in deviance with other previous studies elsewhere [67,68]. This negative relationship is an indication of low tolerance range of bulinid species. Other studies showed no association between snail abundance and water temperature, and it was suggested that this may have been due to the narrow range of temperature [69,70].

The pH of an aquatic habitat is an indicator of the water quality and the extent of pollution [71]. Unpolluted Rivers normally show a near neutral or slightly alkaline pH. The significant positive correlation between pH and some bulinids in this study was in consonance with other studies [72]. However, on the contrary, Levitz and other colleagues [73] have reported that a lower pH (more acidic) was associated with higher snail abundance. In this study, the pH values in all the water bodies was within favourable range for freshwater snail development [49] except the pH values recorded in Ajerogun River in Ibese which was below 6.0 and the pH value of less than 5.5 have been incriminated to be detrimental to the survival of snails species [74] which could be traced to the nature of the sediment.

Land use/land cover changes in some parts of Nigeria have been linked with high schistosomiasis [75,76]. The positive relationship which occurred between snail intermediate hosts species and LULC was expected, as continuous impact of anthropogenic factor on the land surface results in the creation of more suitable habitats for the snail intermediate hosts. Each environmental effect, either occurring as a natural phenomenon or through human intervention, changes the ecological balance and context within which intermediate hosts breed, develop, and transmit diseases. Each species occupies a particular ecological niche and intermediate hosts species are distinct behaviourally and genetically as they adapt to man-made environments. Deforestation, human settlements, commercial developments, road constructions, water control systems and climate change, singly, and in combination, result in global increase in morbidity and mortality due to the emergence of parasitic diseases. The replacement of forests with crop farming, ranching, and raising of small animals can create supportive habitats for parasites and their intermediate hosts [7].

In the observation by Ferguson [77], no significant relationship occurred between the prevalence of schistosomiasis and social connectivity. Besides, disease prevalence may also be influenced by other inter-village movements, notably agricultural activities [78] and commercial development.

The area covered by water bodies increased from 3.72 to 4.51 kilometer, this indicate that, more suitable habitats were being created for the multiplication of freshwater snails. We therefore conclude that increase in areas suitable for the survival of freshwater snails could lead to an increase in water-borne diseases caused by the availability of snail intermediate hosts. However, more study is required in order to understand the risk profile distribution of this snail species. This will, in turn, give more insight into the role of these snails in epidemiology of parasitic diseases.
